# The association of breastfeeding self-efficacy with breastfeeding duration and exclusivity: longitudinal assessment of the predictive validity of the Greek version of the BSES-SF tool

**DOI:** 10.1186/s12884-021-03878-3

**Published:** 2021-06-09

**Authors:** Mary Economou, Ourania Kolokotroni, Irene Paphiti-Demetriou, Christiana Kouta, Ekaterini Lambrinou, Eleni Hadjigeorgiou, Vasiliki Hadjiona, Nicos Middleton

**Affiliations:** 1 Department of Nursing, School of Health Sciences, Cyprus University of Technology, Limassol, Cyprus; 2 St George University of London Medical School at the University of Nicosia, Nicosia, Cyprus; 3 Cyprus Breastfeeding Association – ‘Gift for Life’, Nicosia, Cyprus

**Keywords:** Breastfeeding, Exclusivity, Breastfeeding self-efficacy, Reliability, Validity

## Abstract

**Introduction:**

While breastfeeding self-efficacy (BSES) is an important modifiable determinant of breastfeeding, a structured assessment is not standard practice in Cyprus. We assessed the Greek version of the Breastfeeding Self-Efficacy Scale (BSES-SF), including its predictive validity in terms of Breastfeeding (BF) and Exclusive Breastfeeding (EBF) up to the sixth month.

**Methods:**

A methodological study with longitudinal design among 586 mother-infant dyads, as part of the “BrEaST Start in Life” project. BSES was assessed 24–48 h after birth and at the first month. Breastfeeding status was assessed at the clinic, the 1st, 4th and 6th month. The association between BSES and breastfeeding was estimated in logistic regression models and its diagnostic ability in ROC analysis.

**Results:**

With Mean = 3.55 (SD = 0.85), BSES was moderate, and lower among Cypriot women, primiparas and those who delivered by Cesarean Section (C/S). There was good internal consistency across the 14 items (Cronbach’s α = 0.94) while factor analysis revealed a two-factor structure. BSES scores were higher among mothers who initiated exclusive breastfeeding (M = 3.92, SD = 0.80) compared to breastfeeding not exclusively (M = 3.29, SD = 0.84) and not breastfeeding (M = 3.04, SD = 1.09; *p*-value < 0.001). There was a stepwise association with exclusivity (40.5% in the highest vs 7.9% lowest quartile of self-efficacy). The association between in-hospital BSES and long-term EBF persisted in multivariable models. Women in the upper quartile of BSES at 48 h were more likely to breastfeed exclusively by adjOR = 5.3 (95% CI 1.7–17.1) at the 1st and adjOR = 13.7 (95% CI 2.7–68.6) at the 4th month. Similar associations were observed between self-efficacy at the 1st month and BF at subsequent time-points. High first month BSES (> 3.96 as per ROC) had 58.9% positive and 79.6% negative predictive value for breastfeeding at 6 months which reflects higher sensitivity but lower specificity.

**Conclusions:**

The Greek version of BSES-SF showed good metric properties (construct, know-group, concurrent and predictive validity). In the absence of community support structures or programmes in Cyprus, prevalence of breastfeeding remains low. This suggests a need for policy, educational and community support interventions, including the systematic use of BSES scale as a screening tool to identify those at higher risk for premature BF discontinuation.

**Supplementary Information:**

The online version contains supplementary material available at 10.1186/s12884-021-03878-3.

## Introduction

Breast milk is the optimal source of infant nourishment and addresses physiological and psychological requirements of the newborn during the first months of life. Numerous studies confirm the short- and long-term beneficial effects of breastfeeding for both infant and mother [1, 2]. To achieve optimization of all health benefits, the World Health Organization suggests exclusive breastfeeding (EBF) for the first 6 months and continued breastfeeding (BF) until 2 years of age or beyond [3]. Currently, prevalence of exclusive breastfeeding for infants aged 0–6 months does not exceed 38% [4]. This falls short of the *World Health Assembly Global target for Nutrition* by 2025, which called for an increase in the global rate of exclusive breastfeeding in the first 6 months to at least 50% [5]. A recent publication by Rito at el (2019) suggested that exclusive breastfeeding rates for at least 6 months range from 73.3% in Tajikistan to 10.5% in Italy [6].

Even though the majority of women in Cyprus initiate breastfeeding while at the maternity clinic, in terms of exclusive breastfeeding, Cyprus ranks in the lowest positions in Europe [7]. The *BrEaST start in life* study documented the maternity clinic practices in relation to the implementation of the 10 steps for successful breastfeeding [8] and provided first-time estimates of the prevalence of breastfeeding and exclusive breastfeeding beyond 48 h [9]. The prevalence of exclusive breastfeeding was as low as 18%, even at 48 h, while by the sixth month, one in three women continues breastfeeding but only 1 in 20 exclusively [9], an estimate even lower than previously reported figures. There is an extensive literature on several determinants that contribute to the low breastfeeding rates, including demographic, lifestyle, psychosocial and biomedical factors. In order to tackle the low breastfeeding rates, emphasis should be given to modifiable determinants. Maternal Breastfeeding Self-efficacy has been identified as one of the strongest modifiable predictors of breastfeeding initiation, duration and exclusivity [10, 11]. On the basis of Bandura’ s Self-Efficacy Theory [12], Dennis developed a theoretical framework of the effect of maternal breastfeeding self-efficacy, defined as the mother’s perceived ability to breastfeed her infant, on breastfeeding outcomes [13]. Self-efficacy determines the performance of a specific behavior “*as it reflects of the individual perceptions about their perceived ability, not their actual ability*” [13]. As a cognitive process, a strong sense of self-efficacy results in a positive perception and success promoting thought patterns with regards to the mother’s ability to breastfeed. In contrast, low self-efficacy is more likely to be associated with negative experiences as well as thought patterns and emotional reactions. For example, when a mother expects to fail to breastfeed, she perceives it as discouraging and overwhelming [14, 15]. In turn, this is more likely to result in early breastfeeding discontinuation. The 33-item Breastfeeding Self-Efficacy Scale was developed based on this theoretical model with the aim to identify mothers at risk to discontinue breastfeeding [10]. Due to item redundancy, a shorter 14-item form of the scale with similar psychometric properties was proposed [16]. The BSES-Short Form has been translated and validated in many languages, including Polish [17], Croatian [18], Hong Kong Chinese [19], Turkish [20], Swedish [21], Portuguese [22] and Spanish [23]. It has been extensively used in studies among the general population of mothers or specific population groups; adolescents [24], primiparas [25–27], ethnic minorities [28–30], low income [31] and many more. Only recently, a 9-item exclusive breastfeeding specific self-efficacy scale was developed on the basis of Dennis BFSE-SF scale to assess EBF where breastfeeding is common, with very good psychometric properties [32]. Research evidence suggests a positive predictive association of breastfeeding self-efficacy and breastfeeding initiation, duration and exclusivity [17, 18, 20, 22–24, 31]. Assessment of maternal breastfeeding self-efficacy prenatally or/and postnatally can identify women at high risk of breastfeeding discontinuation and thus in need of breastfeeding support.

The BSES scale, either in its original or short form, is most widely used scale in the literature. Even though the BSES was available in Greek (personal contact with the developer), no study assessing the breastfeeding self-efficacy of Greek or Greek-Cypriot mothers had been identified in the published or grey literature at the time of designing the study protocol. The purpose of this study was to assess some of the metric properties of the Greek version of the Breastfeeding Self-Efficacy Scale - short form (BSES-SF) with a focus on its predictive validity among a sample of women giving birth in Cyprus. Specifically, the objectives of the study were to assess the dimensionality of the scale (factor validity), known-group validity (based on the assumption that self-efficacy is expected to higher in multiparas vs primiparas), concurrent validity (against initiation of breastfeeding at 48 h and exclusivity status) and finally, its predictive validity (against continued breastfeeding up to the sixth month) which even though not always considered in similar studies, it would support its usefulness as a potential screening tool.

## Methods

### Study design

This methodological study with longitudinal design was part of the wider research program “*BrEaST start in life”* aimed at strengthening the evidence-base around breastfeeding in Cyprus. The programme explored various determinants of breastfeeding, including the degree of implementation of the Baby-Friendly Hospital Initiative’s “10 steps for successful breastfeeding”, which form the basis of the National Strategy and Policy of the Cyprus National Breastfeeding Committee. The parent study was a nationwide cross-sectional (at phase I) and longitudinal (phase II) descriptive study. The study design was described in detail previously [9]. In brief, a nationwide sample of mothers was recruited cross-sectionally in the first phase from maternity clinics with the main aim to assess the extent and degree to which the WHO 10 steps are implemented. At phase II, mothers recruited at phase I were followed up prospectively with a telephone interview at three time points: first, fourth and sixth month. This allowed first-time estimates of the prevalence of any and exclusive breastfeeding in Cyprus since the only available official reported statistics simply refer to the first 48 h. The study participants also provided information about a range of potential determinants of breastfeeding at each contact, including breastfeeding self-efficacy, which is the focus of this article.

### Study setting

All maternity clinics in Cyprus were formally invited to participate and maternity wards in state hospitals (5 in total) and 24 of 30 private clinics agreed to participate. A convenience consecutive sample of mother-infant dyads was recruited during stay at the maternity clinics based on pre-defined criteria (see section below). The recruitment period was constant across all sites (6–8 weeks) in order to approximate the correct distribution of births across settings, as there is no official clinic-level record of the number of births in the private sector, which nevertheless accounts for over 70% of births. Trained field workers approached the women between 24 and 48 h after birth and asked them to complete a battery of self-administered questionnaires while waiting outside. Mothers who consented to participate at the next phase of the study, were followed up with a telephone interview. Breastfeeding self-efficacy was assessed in-hospital and at the 1st month telephone follow-up. Infant feeding practices, including breastfeeding and exclusive breastfeeding status, were assessed at all contacts with participants i.e. 48 h, 1st, 4th and 6th month.

### Eligibility criteria

Mothers were eligible to participate if they gave birth to a live infant in the participating clinics during recruitment, irrespective of whether they had a single or multiple pregnancy, they were at least 18 years of age, could read or speak Greek or English, had no health problems precluding them from breastfeeding, as recorded in the medical file and/or communicated to the team by the clinic staff (e.g. bilateral mastectomy, postpartum maternal complications) and were not separated from their infants after birth for medical reason, which would not allow breastfeeding initiation within 1 h, e.g. transferred to NICU at the same hospital or at a different location (low birth weight < 2500 g, gestational period< 37 weeks). The socio-demographic and clinical (e.g. C/S rates) profile of participants was compared to official national figures to assess representativeness of sample. Socio-demographic characteristics of non-participants or participants lost-to-follow up were also used to assess the extent of possible selection bias.

### Measurement tools

At baseline, mothers were asked to complete the Perceived Breastfeeding Self-efficacy scale – short-form (BSES-SF), developed by Dennis & Faux [10]. This was included in a questionnaire pack with consisted of three sections: (a) the WHO/ UNICEF questionnaire - Section 4 [33] on the self-reported experience of the “10 Steps for Successful Breastfeeding”, (b) the breastfeeding self-efficacy scale and (c) socio-demographic information. Participating mothers were asked to provide information on parity, breastfeeding history, intention to breastfeed, lifestyle factors (e.g. smoking, alcohol) and other. At the first month telephone follow-up, breastfeeding mothers completed the BSES-SF.

Information on infant feeding practices was collected at each subsequent contact, such as the type as well as time of introduction and frequency of supplemental feeding including formula, other liquids, solids, medication, vitamin, mineral drops or Oral Rehydration Solution (ORS). Self-reported current status, 24-h recall as well as a retrospective event calendar method were used to estimate the prevalence of breastfeeding (defined as breast milk in addition to any other liquid or food, including formula) and exclusive breastfeeding (defined as no other liquids or solids other than breast milk, given from any source, with the exception of medication, vitamin or mineral drops, Oral Rehydration Solution). The definitions, questions and process for determining breastfeeding status was previously reported in detail [9]. Note that 24 h recall of feeding practices were recorded using a modified version of the Centers for Disease Control and Prevention IFP questionnaire used in the Infant Feeding Practices Study II (IFPS II) in order to take into consideration predominant local practices.

### Breastfeeding self-efficacy scale short form (BSES-SF)

In its short-form, the BSES-SF contains 14 items with a five-point Likert scale response-set. All statement are phrased positively, and begin with the phrase ‘*I can always…*’. The response scale ranges from 1 = not at all confident and 5 = very confident. The theoretical range of the scale is 14–70, with higher scores indicative of higher levels of breastfeeding self-efficacy. Commonly, the total score is calculated by aggregating the responses on all 14 statements. In this study, the average (rather than the overall) score was used i.e. dividing the total score by the number of items. Other than allowing the inclusion of a small number of questionnaires (*N* = 27) for which an answer was not provided on all 14 items, this allows to express the score on a scale of 1–5. There are no set cut-off values for the scale. However, it has been suggested that a 5–10 unit difference (approximately 0.5–1.0 SD) is clinically significant in terms of predicting breastfeeding success in the long-run. A 10-unit difference in the aggregate score would correspond to 0.7 on a 1–5 scale.

### Translation of the tool

The tool was used with permission by the developer who provided an existing Greek translation of the scale, even though at the time of designing the study protocol, no study that have used the Greek version of BSES- or BSES-SF was identified in the literature.. A number of grammatical changes were deemed necessary, after a double forward-backward translation process, in order to improve readability. The main change was in terms of the correct use of the tense for the key verbs to refer to a continuing process (i.e. every time) as also intended by the use of the term “always” in every item and not as an isolated event which often was unintentionally the effect in the use of the wrong tense for the verb in the provided Greek version. A number of other syntactical changes were also deemed necessary to improve its readability, for example “I can always preserve my willingness to breastfeed” was more accurately and succinctly translated as “Μπορώ πάντα να διατηρώ τη θέληση μου να θηλάζω” rather than “Μπορώ πάντα να θέλω να συνεχίζω να θηλάσω” in the original (which backwards translates as “I can always want to continue to breastfeed”, which is not just more awkward to read but does not convey the same meaning. Any necessary changes were identified as a result of the translation process, debated and agreed by consensus among the research team. Alternative versions were discussed during the pilot testing of the tool for readability among 11 mothers who gave birth in Nicosia maternity clinics, before the launch of the nationwide study, after which no further changes were introduced. A study published subsequently (Iliadou et al. 2020) used the tool in a Greek speaking population in Athens. However, the authors of that study state that they used the translated version as provided by the developer without describing whether any changes were introduced. The Greek translation of the tool used in this study is included as an Additional file.

### Ethical considerations

All necessary approvals were obtained from all involved bodies: Cyprus National Bioethics Committee, Research Promotion Committee of the Ministry of Health, which also grants access permission to state hospitals, and equivalently from the administration of all participating clinics. Furthermore, notification was sent to the Commissioner of Personal Data Protection. Separate written consent was obtained for participating at each phase of the study in order to ensure higher participation at baseline. Mothers were informed that participation was volunteer and they could withdraw their participation at any time point of the study. Confidentiality and anonymity were assured.

### Sample size calculations

Sample size calculation and, consequently the period of recruitment, was based on precision analysis with finite population correction (birth cohort around 10,000 annually) to estimate the prevalence of breastfeeding with 95% confidence interval not wider that ±5%, since this was the main aim of the parent study. This was estimated at a minimum required sample size of 370 but it was inflated to secure a sizeable sample at baseline to allow for potential drop-outs in the follow-up phases. The sample size, as estimated above, was also assessed using power analysis in terms of detecting an association between breastfeeding outcomes and a range of determinants of breastfeeding based on the range of estimates observed in previous studies, including breastfeeding self-efficacy. Even though estimates in the literature for the association between breastfeeding self-efficacy and breastfeeding outcomes are not directly comparable, due to different study designs, analytical approaches and length of follow-up, studies commonly report estimates in the magnitude of 2.00 in the odds ratio scale for any breastfeeding, or higher in the case of exclusive breastfeeding, per 5–10 units increase in the aggregate BSES scores. It was estimated a priori that a sample size of 370 provided 90% power to detect an association in the magnitude of 1.7 in the odds ratio scale at the 5% statistical significance level, which was within the range of expected association. A post-hoc power analysis was also performed to estimate the retrospective power of the actual sample size based on observed effect sizes, which were actually larger than originally anticipated. For instance, even with the slightly smallest sample at the 6th month of follow-up, it was estimated that the study had 90% power to detect as little as 0.35 SD difference between two comparison groups of interest, which corresponds to about 4-point difference in the scale. In fact, 5.5–8.5 mean differences were recorded in breastfeeding self-efficacy scores between mothers who did and did not breastfeeding throughout the follow-up period, with the difference becoming larger as time progressed.

### Statistical analysis

Maternal Breastfeeding Self-Efficacy was expressed as a continuous variable (i.e. average score) as well as an ordinal variable (i.e. quartiles of participants based on the score distribution). The choice of using a statistical criterion was preferable due to the lack of generally accepted or uniform cut-off points reported across studies and populations. Thus, the predictive validity of the scale was explored in terms of its association with breastfeeding status in logistic regression models across quartiles of participants with increasing BSES scores. The identification of potential determinants of breastfeeding self-efficacy, including assessing the known-group validity of the scale, were explored in terms of observed differences in mean BSES between subgroups of participants based on their sociodemographic and other characteristics in one-way analysis of variance (ANOVA) and independent t-test as appropriate. Visual assessment and statistical tests were used to assess the symmetry and normality of the distribution of the Breastfeeding Self-efficacy scores at 48 h and 1st month. Bonferroni post hoc comparisons were performed were necessary. Construct validity was evaluated through exploratory factor analysis with a principal components extraction with a varimax rotation to identify the dimensionality of the scale. Reliability was assessed using the Cronbach‘s alpha coefficient for internal consistency. Concurrent and predictive validity of BSES was explored with the investigation of the association of breastfeeding self-efficacy as measured at baseline and at first month with infant feeding practices postpartum. Odds ratios (and 95% CI) of BF/EBF at each time point across quartiles of increasing BSES were estimated in logistic regression models before and after adjusting for important covariates of BSES, as identified in multivariable stepwise linear regression models. The diagnostic ability of the scale was also assessed by calculating the receiver operating characteristics (ROC) curve and examining the area under the curve. The point on the ROC curve with the highest combined Sensitivity and Specificity as indicated by the area under the curve (AUC) was proposed as the potential cut-off point of the scale. G power was used for sample size calculations and SPSS for Windows Version 21(SPSS Inc., Chicago, IL, USA) was used for the analyses.

## Results

### Participant characteristics

The baseline sample consisted of 586 mother-infant dyads (response rate 73.5% among the 797 consecutive sample eligible to participate), approximating the expected national distribution of births across districts and 70:30 split between private and public sector (not presented in detail). Of those, 372 (response rate: 63.5%), 383 and 340 mothers respectively participated at the first, fourth and sixth month follow-up. The total number of mothers who completed the BSES-SF scale at 48 h and 1st first month was 504 and 284 respectively, since with a few exceptions, those who did not initiate breastfeeding or were not breastfeeding at the time of assessment did not respond to the scale. As shown in Table 1, the majority of women were aged 25–29 years (46.3%) and 30–34 (21.0%). Only 6.3% of participating mothers were older than 35 years of age. The percentage in the sample who were of Cypriot origin was 77.2%, which is consistent with official statistics. In terms of educational attainment, 57.3% had either undergraduate or postgraduate tertiary education. For 48.9% of mothers, this was their first child. Among the rest, 44.7% reported previous breastfeeding experience. As reported previously [9], the prevalence of breastfeeding at 48 h was 84.3% (95% CI 81.4–87.3%). Even though the true prevalence of exclusive breastfeeding was only 18.8% (95% CI 15.6–21.9%), 81.9% of mothers reported their intention to breastfeed exclusively and 76.6% to do so for at least 6 months. It is of note that as many as 55.8% delivered by C/S, which is consistent with officially published data.

**Table 1:** Participants’ characteristics and perceived breastfeeding self-efficacy by socio-demographic and other characteristics

	*N (%)*^a^	BSES-SF Mean (SD) at 48 h	*p*‡	*N (%)*^a^	BSES-SF Mean (SD) at 1st month	*p*‡
**Age**						
18–24	**126 (25.3)**	3.43 (0.85)	**0.45**	**62 (23.1)**	4.04 (0.68)	**0.94**
25–29	**231 (46.4)**	3.41 (0.90)		**129 (47.9)**	4.00 (0.80)	
30–34	**108 (21.7)**	3.31 (0.96)		**59 (21.9)**	3.96 (0.91)	
≥ 35	**33 (6.6)**	3.59 (0.90)		**19 (7.1)**	4.06 (0.66)	
**Education**						
At most secondary	**208 (42.6)**	3.41 (0.90)	**0.03**	**116 (44.3)**	4.03 (0.77)	**0.75**
Undergraduate	**162 (33.2)**	3.51 (0.91)		**89 (33.9)**	3.99 (0.76)	
Postgraduate	**118 (24.1)**	3.22 (0.85)		**57 (21.8)**	3.94 (0.87)	
**Marital Status**						
Married/Cohabiting	**484 (97.4)**	3.39 (0.90)	**0.34**	**259 (97.0)**	4.00 (0.79)	**0.69**
Other	**13 (2.6)**	3.63 (0.86)		**8 (3.0)**	4.10 (0.69)	
**Employment Status**						
Full Time	**324 (65.6)**	3.35 (0.87)	**0.16**	**170 (63.9)**	3.94 (0.82)	**0.18**
Part Time	**55 (11.1)**	3.54 (0.94)		**33 (12.4)**	4.04 (0.68)	
Unemployed	**115 (23.3)**	3.49 (0.92)		**63 (23.7)**	4.16 (0.75)	
**Monthly Family Net Income**						
< = €1500	**202 (45.2)**	3.44 (0.90)	**0.46**	**119 (48.8)**	4.05 (0.72)	**0.15**
€1501- €3000	**178 (39.8)**	3.33 (0.86)		**86 (35.2)**	3.88 (0.86)	
> = €3001	**67 (15.0)**	3.43 (0.94)		**39 (16.0)**	4.09 (0.82)	
**Country of Origin**						
Cypriot	**380 (77.2)**	3.29 (0.86)	**< 0.001**	**194 (73.5)**	3.95 (0.78)	**0.03**
Not Cypriot	**112 (22.8)**	3.83 (0.88)		**70 (26.5)**	4.19 (0.77)	
**Type of Birth**						
Vaginal	**223 (44.2)**	3.57 (0.87)	**< 0.001**	**135 (49.1)**	4.17 (0.73)	**0.002**
C/S w/t Gen Anesthesia	**213 (42.3)**	3.23 (0.89)		**93 (33.8)**	3.93 (0.81)	
C/S w Gen Anesthesia	**68 (13.5)**	3.37 (0.93)		**47 (17.1)**	3.72 (0.76)	
**Parity**						
First child	**246 (48.9)**	3.17 (0.81)	**< 0.001**	**140 (49.3)**	3.81 (0.79)	**< 0.001**
Multiparous w/t previous BF experience	**32 (6.4)**	2.78 (1.01)		**7 (2.5)**	3.70 (1.18)	
Multiparous w/ previous BF experience	**225 (44.7)**	3.74 (0.84)		**137 (48.2)**	4.23 (0.67)	
**Intention to breastfeed exclusively**						
Yes	**405 (81.9)**	3.54 (0.83)	**< 0.001**	**237 (88.4)**	4.05 (0.76)	**0.002**
No	**92 (18.5)**	2.80 (0.94)		**31 (11.5)**	3.60 (0.84)	
**Intention to breastfeed exclusively for 6 months**						
Yes	**377 (76.6)**	3.57 (0.82)	**< 0.001**	**225 (84.9)**	4.08 (0.75)	**< 0.001**
No	**115 (23.4)**	2.85 (0.93)		**40 (15.1)**	3.53 (0.85)	

‡*p*-values as estimated using independent sample t-test or one-way ANOVA, as appropriate

^a^Total number of breastfeeding mothers who completed the BSES-SF scale at 48 h and 1st first month: *N* = 504 and *N* = 284 respectively. Participants with missing socio-demographic information were excluded from the statistical analysis. The percentage of missing values was generally low and ranged between 0 and 7% (*N* = 488–504 and 262–284 respectively), with the exception of family income (*N* = 447 and 244 respectively)

### Internal consistency of BSES-SF

The internal consistency of the scale was estimated by Cronbach’s α coefficient at 0.94, which is identical to the one reported in the original study [16]. There was no increase by more than 0.1 in the alpha coefficient in response to the deletion of any items. The inter-item correlations ranged between 0.26 to 0.82, with a mean of 0.55.

### Breastfeeding self-efficacy by socio-demographic characteristics

The mean breastfeeding self-efficacy score was 3.55 (SD = 0.85, median = 3.57, IQR = 2.71–4.04, range 1–5) within the first 48 h and 4.01 (SD = 0.79, median = 4.14, IQR = 3.57–4.64, range = 1–5) at the first month assessment. Table 1 shows mean levels (SD) of BSES scores according to sociodemographic characteristics. A statistically significant difference in mean BSES scores was observed by mode of delivery. Mothers who had vaginal delivery were more likely to report higher levels of BSES at 48 h (3.57, SD = 0.87) compared to those who gave birth by C/S with general anesthesia (3.37, SD = 0.93) and C/S with regional (not general) anesthesia (3.23, SD=SD: 0.89; *p*-value< 0.001). Even though BSES scores were generally higher for all by the first month, a significant difference across different types of delivery was still apparent, corresponding to a moderate effect size in the magnitude of 0.5 SD. Mothers who intended to EBF for 6 months reported significantly higher levels of BSES from the first 48 h (M = 3.37, SD = 0.82 vs M = 2.85, SD = 0.93; *p*-value< 0.001). With regards to nationality, Cypriot mothers (M = 3.29, SD = 0.86) were more likely to report lower levels of BSES than non-Cypriot mothers (M = 3.83, SD = 0.88; p- value< 0.001). This difference appeared smaller by the first month, but remained statistically significant (*p*-value = 0.026).

Surprisingly, mothers with postgraduate education had the lowest BSES scores (M = 3.22, SD = 0.85) compared to both mothers with University or College education (M = 3.51, SD = 0.91) as well as those with at most secondary education (M = 3.51, SD = 0.91; *p*-value = 0.025). By the first month, this difference was no longer apparent and the mean scores of the three groups appear similar.

### Validity

#### Factor validity

The construct validity of the BSES-SF was assessed in Exploratory Factor Analyses – see Table 2. The correlation matrix was adequate with the majority of the correlation coefficients exceeding 0.3. The Kaiser–Meyer–Olkin coefficient for sampling adequacy (KMO) was 0.96, which is above the recommended value of 0.60 and the Bartlett’s test of Sphericity was significant (*p*-value< 0.001), both suggesting that the data are appropriate for factor analysis. Based on the default criterion of eigenvalues greater than 1.0, the analysis yielded a one-factor solution even though the scree plot was suggestive of a two-dimensional structure*.*

**Table 2:** Principal component analysis of the BSES-SF scale

	I can always…	Component 1	Component 2
1	Determine that my baby is getting enough milk		0.803
2	Successfully cope with breastfeeding like I have with other challenging tasks		0.763
3	Breastfeed my baby without using formula as a supplement		0.618
4	Ensure that my baby is properly latched on for the whole feeding		0.752
5	Manage the breastfeeding situation to my satisfaction		0.775
6	Manage to breastfeed even if my baby is crying		0.630
7	Keep wanting to breastfeed	0.784	
8	Comfortably breastfeed with my family members present	0.641	
9	Be satisfied with my breastfeeding experience	0.721	
10	Deal with the fact that breastfeeding can be time-consuming	0.788	
11	Finish feeding my baby on one breast before switching to the other breast	0.574	0.512
12	Continue to breastfeed my baby for every feeding	0.687	
13	Manage to keep up with my baby’s breastfeeding demands	0.653	
14	Tell when my baby is finished breastfeeding	0.617	
**% of variance explained**		**58.5%**	**7.48%**
**Cronbach’s alpha coefficient**		**0.923**	**0.911**

Table 2 depicts the principal component analysis of the BSES scale. The first component included eight items, explaining 33.5% of the variance. With a few exceptions (e.g. “*Tell when my baby is finished breastfeeding*”), most items were more likely to tap on Breastfeeding Self-efficacy in terms of the cognitive aspects, such as “*Keep wanting to breastfeed*”, “*Comfortably breastfeed with my family members present*” and “*Deal with the fact that breastfeeding can be time-consuming*”. The second component included the remaining six items, and explained 32.5% of the variance. These were more likely to tap on the technical aspects of breastfeeding management. For example, “*Determine that my baby is getting enough milk*”, “*Ensure that my baby is properly latched on for the whole feeding*” and “*Manage to breastfeed even if my baby is crying*”. One of the items (“*Finishing feeding my baby on one breast before switching to the other*”) cross-loaded in both components with similar factor loadings.

The tool appears to be tapping on two different aspects of breastfeeding self-efficacy. While these two aspects have been described previously in the literature, calculating an overall score seems to be the most standard approach. Thus, for comparability, the overall score was used for further analyses.

#### Known-group validity

According to Bandura, previous experiences have a significant influence on self-efficacy [12]. Thus, multiparous mothers with previous breastfeeding experience would be expected to be more likely to have higher BSES compared with primiparous mothers. A known-group comparison analysis was conducted to assess this assumption. As shown in Table 1, mothers with previous breastfeeding experience were more likely to report higher BSES (M = 3.74, SD = 0.84) compared to both primiparas (M = 3.17, SD = 0.81) as well as multiparas without BF experience (M = 2.78, SD = 1.01; < 0.001), who appear to have the lowest levels of breastfeeding self-efficacy, even compared to primiparas. The observed difference is statistically as well as clinically significant, as it represents a 0.7 SD difference in mean scores. This difference was still apparent at the first month after discharge.

In a stepwise linear regression analysis, parity along with intention to BF, initiation of BF, non-Cypriot background, vaginal delivery and tertiary, but not postgraduate, education were the only ones associated with BSES, explaining 24% of the variance 48 h postnatally - results not shown in a Table. In fact, parity (β = 0.414, 95% CI: 0.264, 0.564; *p*-value = < 0.001) and intention to EBF (β = 0.642, 95% CI: 0.450–0.834; *p*-value< 0.001) showed the strongest associations with BSES. Other variables predictive of in-hospital BSES scores were initiation of BF (β = 0.498, 95% CI: 0.133, 0.862; *p*-value = 0.008), non-Cypriot background (β = 0.422, 95% CI: 0.240, 0.604; *p*-value< 0.001), vaginal delivery (β = 0.273; 95% CI: 0.120, 0.427; *p*-value = 0.001) and university, but not postgraduate, education (β = 0.182; 95% CI: 0.022, 0.342; *p*-value = 0.026).

#### Concurrent and predictive validity

Concurrent and predictive validity of the scale was evaluated by assessing the differences in BSES scores according to breastfeeding initiation and status at 48 h and thereafter up to the sixth month of the infants’ life. BSES scores during the first 48 h were highest among mothers who initiated exclusively breastfeeding while at the clinic (M = 3.92, SD = 0.80) compared to those who were breastfeeding not exclusively (M = 3.29, SD = 0.84) and those not breastfeeding (M = 3.04, SD = 1.09; *p*-value < 0.001) – see Table 3. A similar stepwise pattern of association with breastfeeding status and BSES scores was observed across all follow-up phases of the study, irrespective of whether the analysis looked at the in-hospital or the 1st month assessment of BSES. Consistently, mothers who were still exclusively breastfeeding at a specific time-point of investigation were those who reported higher on average BSES scores (in-hospital or at 1st month), with progressively lower mean scores observed among mother who were either breastfeeding but not exclusively or not breastfeeding by that point.

**Table 3:** In-hospital and 1st month BSES mean scores by breastfeeding status at 48 h and at first, fourth and sixth month

	Mean (SD) BSES-SF score					
	N	48 h	*p*-value¥	N	1st month	*p*-value¥
**According to BF status**						
**At 48 h**						
EBF	104	3.92 (0.80)	< 0.001		–	
BF	365	3.29 (0.84)			–	
Non-BF	35	3.04 (1.09)			–	
**At 1st month**						
EBF	58	3.85 (0.86)	< 0.001	64	4.39 (0.66)	< 0.001
BF	193	3.49 (0.84)		206	3.95 (0.75)	
Non-BF	70	2.96 (0.93)		14	3.23 (0.74)	
**At 4th month**						
EBF	45	3.96 (0.67)	< 0.001	40	4.45 (0.54)	< 0.001
BF	114	3.65 (0.82)		105	4.29 (0.65)	
Non-BF	184	3.15 (0.92)		111	3.60 (0.80)	
**At 6th month**						
EBF	18	3.90 (0.76)	< 0.001	19	4.55 (0.35)	< 0.001
BF	97	3.73 (0.79)		88	4.32 (0.62)	
Non-BF	213	3.23 (0.92)		142	3.71 (0.81)	

¥*p*-value of one-way ANOVA

#### Association of BSES with continuation and exclusivity of breastfeeding

**Fig. 1:**
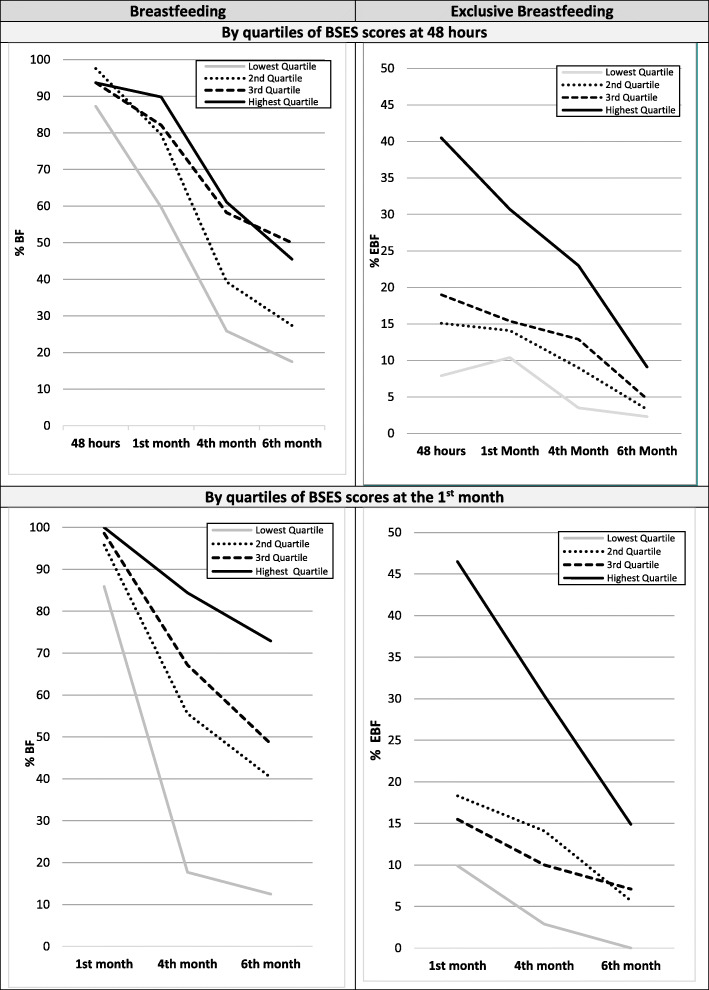
Prevalence of breastfeeding and exclusive breastfeeding by quartiles of increasing BSES scores at 48 h and 1st month

To investigate the association of BSES with BF status, we categorized participating mothers based on their BSES scores at 48 h and first month into quartiles. At 48 h, for example, the lowest quartile includes the quarter of women with the lowest scores (range: 1.00–2.71) and the upper quartile, those with the highest scores (range: 4.07–5.00). While not large differences were observed in terms of initiation of breastfeeding according to BSES, as shown in Fig. 1, there appears to be a clear stepwise pattern of association of BSES with EBF and BF continuation up to the sixth month. For instance, among the quartile of mothers with the highest BSES scores, as many as 40% initiated exclusive breastfeeding during their stay at the maternity clinic. In contrast, among the quartile of mothers with the lowest BSES scores only 7.9% initiated EBF (*p*-value< 0.001). The prevalence of EBF for the two middle groups appeared in-between with 15.1 and 19.0%, respectively. At the first month, among mothers at the upper quartile of in-hospital BSES scores about three times as many as those in the lower quartile were exclusively breastfeeding (30.7% vs 10.4%) and about twice as many as those in the second and third quartile (30.7% vs 14.1% vs 15.4%, respectively). Differences in the prevalence of EBF at the fourth month widened further and only appear to converge by the sixth month, due to a sharper decline in the prevalence of EBF among mothers with the highest in-hospital BSES scores. Similar stepwise patterns were observed when using BSES as reported at the first month to track BF and EBF continuation beyond the first month.

Table 4 presents the odds of BF and EBF according to quartiles of increasing levels of in-hospital or 1st-month BSES as estimated in multivariable logistic regression models. The clear stepwise pattern of association of BF outcomes with BSES irrespective of whether measured at 48 h or 1st month is apparent even after adjusting for potential confounders such as parity, mode of delivery and social position. For instance, a stepwise increase was observed in the odds of EBF initiation as well as at all other timepoints across quartiles of women with increasing levels of BSES at 48 h. Mothers in the second and third quartile were about two to three times more likely to initiate EBF compared to those in the lowest quartile, while the likelihood of women at the upper quartile to initiate exclusive breastfeeding is 8-times higher (ΟR = 7.89, 95% CI: 3.77–16.49; *p*-value< 0.001). In the multivariable model, the association appears even stronger with adjOR = 9.94 (95% CI: 3.72–26.58; *p*-value< 0.001) in the upper quartile. In relation to EBF continuation, an association with BSES scores at 48 h was observed throughout the study period, attenuating slightly at the sixth month, possibly due to the small number of women exclusively breastfeeding by that point. Even though fewer than 10% of the mothers at the upper quartile were exclusively breastfeeding at the sixth month, this figure was still about four, three and two times higher compared to the respective figure observed among mothers at the lowest (2.3%), the second (3.3%) and the third quartile (4.7%) of BSES scores respectively. The observed stepwise pattern across increasing quartiles of BSES appeared consistent at all time points of investigation irrespective of whether the analysis looked at 48-h or 1stmonth BSES scores.

**Table 4:** Odds ratios of breastfeeding and exclusive breastfeeding at 48 h, 1st, 4th and 6th month by quartiles of participants with increasing breastfeeding self-efficacy scores measured at 48 h and at 1st month, as estimated in univariable and multivariable logistic models

	Based on 48 h assessment of BSES		Based on 1st month assessment of BSES		Based on 48 h assessment of BSES		Based on 1st month assessment of BSES	
	Exclusive breastfeeding				Breastfeeding, even if not exclusively			
	Unadjusted OR (95% CI)	Adjusted OR^b^ (95% CI)	Unadjusted OR (95% CI)	Adjusted OR^b^ (95% CI)	Unadjusted OR (95% CI)	Adjusted OR^b^ (95% CI)	Unadjusted OR (95% CI)	Adjusted OR^b^ (95% CI)
*At 48 h*								
Lower Quartile	1.00	1.00	**–**	–	1.00	1.00	–	–
2nd Quartile	2.06 (0.9, 4.6)	2.15 (0.8, 5.8)	–	–	5.96 (1.7, 21.0)	2.92 (0.7, 12.0)	–	–
3rd Quartile	2.73 (1.3, 6.0)	3.10 (1.2, 8.2)	–	–	2.15 (0.9, 5.2)	1.67 (4.7, 5.9)	–	–
Upper Quartile	7.89 (3.8, 16.5)	9.94 (3.7, 26.5)	–	–	2.15 (0.9, 5.2)	2.20 (0.5, 9.2)	–	–
*At 1st month*^a^								
Lower Quartile	1.00	1.00	1.00	1.00	1.00	1.00	N/A	N/A
2nd Quartile	1.42 (0.5, 3.7)	1.52 (0.5, 5.2)	2.05 (0.8, 5.5)	1.44 (0.5,4.6)	2.61 (1.3, 5.3)	3.18 (1.2, 8.4)		
3rd Quartile	1.57 (0.6, 4.1)	2.08 (0.6, 7.2)	1.68 (0.6, 4.6)	0.78 (0.2, 2.8)	3.08 (1.5, 6.4)	3.51 (1.2, 9.9)		
Upper Quartile	3.82 (1.6, 9.0)	5.31 (1.7, 17.1)	7.94 (3.2, 19.7)	6.05 (2.1, 17.6)	5.92 (2.6, 13.5)	3.92 (1.3, 11.9)		
*At 4th month*								
Lower Quartile	1.00	1.00	1.00	1.00	1.00	1.00	1.00	1.00
2nd Quartile	2.74 (0.7, 10.7)	4.12 (0.8, 22.1)	5.57 (1.2, 26.5)	3.61 (0.6, 20.8)	1.85 (1.0, 3.6)	2.06 (1.0, 4.4)	5.80 (2.6, 13.2)	7.06 (2.6, 19.0)
3rd Quartile	4.11 (1.1, 15.3)	4.28 (0.8, 22.4)	3.78 (0.8, 18.9)	2.84 (0.5, 17.4)	3.99 (2.1, 7.7)	3.75 (1.7, 8.5)	9.48 (4.2, 21.7)	15.6 (5.4, 45.2)
Upper Quartile	8.26 (2.4, 28.6)	13.66 (2.7, 68.6)	14.88 (3.3, 66.5)	9.54 (1.8, 51.3)	4.49 (2.4, 8.5)	4.22 (1.9, 9.4)	25.04 (9.8, 63.9)	28.40 (9.2, 87.3)
*At 6th month*^a^								
Lower Quartile	1.00	1.00	N/A	N/A	1.00	1.00	1.00	1.00
2nd Quartile	1.45 (0.2, 8.9)	1.77 (0.2, 21.6)			1.78 (0.8, 3.8)	2.11 (0.9, 5.1)	4.73 (1.9, 11.6)	5.81 (1.9, 17.3)
3rd Quartile	2.10 (0.4, 11.8)	1.74 (0.1, 21.4)			4.71 (2.3, 9.8)	5.14 (2.1, 12.5)	6.58 (2.7, 16.0)	9.81 (3.1, 30.9)
Upper Quartile	4.25 (0.9, 20.2)	8.90 (0.9, 86.9)			3.93 (1.9, 8.0)	3.64 (1.5, 8.7)	18.81 (7.4, 48.0)	21.55 (6.9, 67.5)

^a^*N/A:* At the sixth month, none of the mothers in the lowest quartile of BSES scores were breastfeeding exclusively. Similarly, all the mothers in the upper quartile of BSES at the first month were breastfeeding, while the sample of non-breastfeeding mothers who completed the BSES at this time point was small; thus, models could not be estimated fully

^b^Adjusted for maternal age, educational attainment, marital status, employment status, family income, parity, country of origin, mode of delivery, previous BF experience, as well as BF at 48 h in models of breastfeeding outcomes beyond 48 h

#### Diagnostic ability of the tool

Table 5 shows the results of the ROC analysis for the predicting ability of the BSES – SF tool to identify mothers likely to successfully continue breastfeeding in the long term. In terms of diagnostic ability, the tool appears to perform better when used at the first month and less well when used at 48 h in terms of predicting BF continuation at 4th and 6th month. For instance, at a cut-off value of 3.96 (which appears to correspond to the highest quartile of scores as observed in this study), the Sensitivity and Specificity of the BSES-SF at 1st month for BF continuation at 4th month is 79.7 and 63.7%, respectively. The positive and negative predictive value are 74.8 and 71.3%, respectively.

**Table 5:** Receiver operating characteristics (ROC) analysis of breastfeeding continuation at 4th and 6th month as measured by the BSES-SF at 48 h and 1st month

	Breastfeeding at 4th month		Exclusive breastfeeding at 4th month^a^		Breastfeeding at 6th month	
	BSES at 48 h	BSES at 1st month	BSES at 48 h	BSES at 1st month	BSES at 48 h	BSES at 1st month
AUC (SE)	0.666 (0.035)	0.779 (0.030)	0.696 (0.042)	0.707 (0.045)	0.646 (0.036)	0.755 (0.032)
Optimal cut-off	3.40	3.96	3.96	3.96	3.40	3.96
Sensitivity (%)	70.3	79.7	63.9	91.7	73.0	81.0
Specificity (%)	58.8	63.7	70.0	44.8	55.3	56.1
Positive predictive value (%)	59.7	74.8	24.6	22.4	48.0	58.9
Negative predictive value (%)	68.5	71.3	93.1	97.4	79.0	79.6

^a^The number of mothers who breastfed exclusively at the 6th month was too small to allow meaningful estimation of the ROC model

## Discussion

### Breastfeeding self-efficacy among mothers in Cyprus

Against a generally low prevalence of breastfeeding among mothers in Cyprus, this study showed that low self-efficacy in the early period is associated with non-exclusivity and earlier discontinuation of breastfeeding. With a mean score of 3.55 (on a 1–5 scale), breastfeeding self-efficacy among women in Cyprus was only moderate. If expressed as a sum (instead of average) score, it corresponds to a score of 49.7 (theoretical range: 14–70). With a few exceptions [19, 25, 34], this is lower than what is commonly reported among other populations in the international literature using the same tool (16–18, 20, 22, 36].

### Dimensionality and internal consistency of the BSES-SF scale

The observed internal consistency of the BSES-SF items was consistent with the original [16] as well as most similar studies in the literature. The BSES-SE seems to be tapping on two aspects of self-efficacy, namely breastfeeding technique and intrapersonal thoughts. Both the original study [16] as well as other translated versions of the scale [17, 18, 20, 22, 34] identify the scale as unidimensional. Our findings are in agreement with a recently published study by Brandão et al. among Portuguese pregnant women which found a similar two-dimensional structure of the BSES-SF scale [35]. In that study, the second component explained only 7.6% of the variance, compared to 53.2% for the first component, whereas in the present study, the percentage of variance explained by the two components was more equally distributed.

### Concurrent and predictive validity of the BSES-SF

As expected, large differences were observed in terms of in-hospital BSES-SF scores according to breastfeeding status, demonstrating the concurrent and predictive validity of the tool. This finding is not surprising and, with a few exceptions [29, 36], it is in agreement with the majority of previous studies. However, only a few studies investigated the association of BSES with BF/EBF up to the sixth month [19, 25–28, 36, 37] as studies commonly investigate the association of BSES with BF within shorter time periods [15, 30, 37–42]. Similar, if not even larger differences were observed between BF/EBF and breastfeeding self-efficacy as reported at the 1st month, and this is also consistent with studies which measured BSES postnatally [19, 25, 27, 28, 36, 43].

The ROC analysis showed that the in-hospital BSES-SF scale at a cut-off value of 3.6 (corresponding to a sum score of 50.4) has acceptable diagnostic ability that a mother would still breastfeed at the 4th month and 6th month. A study by Ip et al. among Hong Kong Chinese mothers found that the BSES-SF at a cut-off value = 45.5 during hospital stay (48–72 h) could be used as a screening tool to identify mothers most likely to discontinue breastfeeding before 6 months with Sn = 73%, Sp = 73%, PPV = 92% and NPV = 42% [19]. In the present study, the predictive value of the scale appeared somewhat better when BSES was assessed at the first month, rather than within the first 48 h. The sensitivity and specificity of the 1st month BSES-SF to identify breastfeeding continuation at 4 months were 79.7 and 63.7% respectively. The sensitivity of the 1st month assessment for breastfeeding at 6 months is very good (81%); however the specificity is average (56.1%) but not surprising as only one in 20 women breastfeed exclusively at 6 months. The positive predictive value of a relatively high score at the first month is 74.8 and 58.9% for breastfeeding at the 4th and 6th month respectively. Thus, one in four and one in two women with high scores will be false positives, and they will discontinue breastfeeding suggesting that several other factors are at play. In terms of the negative predictive value, it is encouraging that a relatively low BSES score at the first month will correctly identify 63.7 and 56.1% of the women who will discontinue breastfeeding by the 4th and 6th month respectively.

### Sociodemographic characteristics and breastfeeding self-efficacy

No differences were observed in in-hospital breastfeeding self-efficacy in relation to maternal demographic characteristics [16–18, 22, 23, 31], with the exception of educational attainment. In fact, mothers with postgraduate education appear to have the lowest breastfeeding self-efficacy levels. This comes in contrast with other findings that suggest a positive relationship between breastfeeding self-efficacy and maternal education [18]. This association diminished by the first month. This may suggest that mothers with higher education are more likely to be aware of the difficulties risen during BF initiation which might result to negative beliefs towards their perceived ability to initiate BF, but are more likely to seek support and overcome the challenges in the long run. In fact, this is consistent with the fact that mothers with postgraduate education appear to have the largest increase in breastfeeding self-efficacy between the two time-points. This also appears consistent with the finding, that, even though there was no difference in the likelihood to initiate exclusive breastfeeding according to educational attainment, in the long term those with the highest educational attainment were 1.8-times (1st month), 2.3-times (4th month) and 3.7-times (6th month) more likely to be exclusively breastfeeding compared to mothers with primary or secondary education – results not shown in detail. It is also interesting to note that Cypriot women appear to have lower on average breastfeeding self-efficacy than non-Cypriot women. The extent to which this is reflective of differences in breastfeeding culture or other breastfeeding determinants between the two groups is not clear. Consistent with previous studies [15, 44–48], mothers with previous breastfeeding experience are more likely to report higher breastfeeding self-efficacy. This results in a higher likelihood of successful BF initiation, continuation and exclusivity [15, 45, 49]. However, there is evidence to suggest that a negative or neutral previous experience may affect breastfeeding self-efficacy negatively [50]. This study did not explore the characteristics of the previous or current experience, which may determine the continuation of the behaviour [51, 52].

Intention to breastfeed was also associated with breastfeeding self-efficacy [45, 48]. This might be explained by the fact that mothers that intent to BF are more likely to be aware about the benefits of exclusive breastfeeding, had attended antenatal educational sessions [48] and seeked formal or informal support [48, 53]. In a recent study, Kronborg et al. found that both intention and self-efficacy are significant mediators of EBF and BF duration even among second-time mothers. It is interesting to note that in this study, even though the actual prevalence of exclusive breastfeeding was only 18.8% at 48 h, 73.2% of mothers reported their intention to breastfeed exclusively [54]. The present study also confirmed the association between breastfeeding self-efficacy and mode of delivery, with mothers who gave birth vaginally having higher levels of breastfeeding self-efficacy [16, 45, 55]. There is evidence to suggest that intention to breastfeed is lower among mothers who give birth by C/S [56]. Furthermore, there is evidence to suggest that women who give birth by C/S are less likely to experience or request the implementation of “good practices” [57]. Experience of the “10 Steps to Successful Breastfeeding” are thought to facilitate the development of breastfeeding skills [58] and thereafter the strengthening of breastfeeding self-efficacy [50, 55], which in turn strengthens maternal commitment to breastfeed [59]. In addition, women who deliver by C/S are more likely to experience breastfeeding difficulties [56] including latching difficulties, perceived lack of infant satiation and perceived lack of infant interest towards breastfeeding [60]. Early BF initiation within 1 h after birth [50], skin-to-skin [61] and rooming-in [50], all of which there is evidence to suggest are not widely implemented in Cyprus [8] have all been positively associated with higher breastfeeding self-efficacy levels. This is consistent with the finding that women who had a C/S without general anesthesia have somewhat higher levels of breastfeeding self-efficacy than those that gave birth with general anesthesia.

### Strengths and limitations

This is the first study to evaluate the psychometric properties of the Greek version of the BSES-SF and describe the breastfeeding self-efficacy of women giving birth in Cyprus. A clear strength is the longitudinal design which facilitated the assessment of feeding practices over the first 6 months, as an indicator of the predictive validity of the tool, avoiding the recall bias of a retrospective design. In fact, it is among a few studies that measured the predictive validity of BSES measured on two occasions on BF duration and exclusivity up to the sixth month, suggesting that the BSES -SF can be a useful tool for the identification of mothers who are more likely to succeed their breastfeeding goal. Even though a number of private clinics opted to self-exclude from the study, the generalizability of findings, at least in a national context, is supported by the fact that the sample is largely representative of the cohort of mothers giving birth in Cypriot maternity clinics. With a response rate of 73.5% at baseline and 63.5% at follow-up, selection bias cannot be ruled out and it is likely that women who intended to breastfeed might be overrepresented in the sample. Even so, the prevalence of exclusive breastfeeding was particularly low while the observed variability in BSES scores among mothers in Cypriot maternity clinics is within the range, if not somewhat higher, than the variability observed in populations elsewhere, since a SD of 0.85 (on a 1–5 scale) corresponds to a SD of 12 on a 14–70 scale. It is also acknowledged that the association between BF and BFSE is likely to be bidirectional (i.e. successful BF establishment positively influences BFSE in the longrun). Like previous similar studies, this study looked at the extent to which BSES is predictive of breastfeeding outcomes in the long term. Ever though the literature is limited with regard to the potential bi-directional association between BF and BSES, there is evidence to suggest that successful early initiation of BF within the first hour after birth, when acknowledged as a positive personal experience, might be associated with higher BFSE levels at first week postpartum [50]. Finally, intention to breastfeed was measured only with a single-item and other potential covariates related to motivation (e.g. beliefs and attitudes related to breastfeeding, maternal personality characteristics, etc) have not been considered.

### Implications for research and practice

In line with the aims, this study used only quantitative methods to explore breastfeeding self-efficacy and subsequent breastfeeding outcomes. However, future studies should focus on an in-depth exploration of the perceptions and attitudes of women in Cyprus with regards to breastfeeding, and perceived reasons for premature discontinuation using qualitative methods. Further research is also required to disentangle the bidirectional association of BSE and BF since personal experience of BF, and the extent to which this is negative or positive, is likely to be the most important source of self-efficacy. A systematic and structured assessment of BSES is not standard practice while the mother is at the clinic, let alone after discharge since in Cyprus there is no continuation of care in the postnatal period. As this study suggests, the BSES could be adopted in clinical practice as a screening tool to facilitate the identification of the mothers at higher risk to discontinue breastfeeding prematurely. However, for this to be effective, it is important to reconsider the current structure of maternal health care services in the community, either through the widening of existing roles or the establishment of new roles such as Community Midwifery. Future research should be focused on the development and the evaluation of breastfeeding community support programmes which aim to enhance maternal breastfeeding self-efficacy. These programmes could include both formal and peer mother-to-mother support groups [62–64]. A number of intervention studies have been designed based on self-efficacy theory and/or investigated the effect of breastfeeding education and/or support programmes explicitly through the enhancement of self-efficacy [65, 66]. For instance, a pre- and post-test experimental study with the participation of 74 Chinese primiparas [65], showed significant differences in BSES between the intervention and the control group at 4 and 8 weeks after birth, while enhancement of BSES in this period was found to be significantly higher in the intervention group. The study showed that the positive impact of the intervention on BF duration and exclusivity, was mediated by the enhancement of BSES. However, a number of breastfeeding self-efficacy studies did not show a positive effect on breastfeeding outcomes [67] or the observed effect was short of statistical significance [68, 69].

As in many European countries, Cyprus has developed mechanisms and initiatives for the support, protection and promotion of BF, including BF policies and strategies [70]. However, it has not yet proceeded to their full integration, implementation or harmonization within the national health system, resulting to the lack of effective breastfeeding promotion actions. Even though the Cyprus Ministry of Health has launched a call for the Baby Friendly Initiative, through the National Breastfeeding Committee, up to date, no hospital, public or private, in Cyprus has moved along with the Baby friendly initiative certification process. Originally a number of clinics had expressed an interest and promoted their intention publicly, a long time has ensued since the original call. This has recently led the National Breastfeeding Committee to assign to an ad hoc committee of practitioners and academics the task of designing the methodology and developing the necessary resources in order to move the process along and support interested hospitals in the process (personal communication). In the meantime, the fragmented implementation of the “10 Steps for Successful Breastfeeding” [8] and in particular the lack of institutionalized community support for breastfeeding mothers (corresponding to Step 10) continue to sustain conditions not contusive to promoting breastfeeding.

## Conclusions

The Greek version of the BSES-SF showed good metric properties and it can be considered a valid and reliable measure of breastfeeding self-efficacy among new mothers in Cyprus. Concurrent and predictive validity of the scale was supported by the observed association of BSES with breastfeeding exclusivity at 48 h and with breastfeeding outcomes at the first, fourth and sixth month of the infants’ life. The adoption of the BSES scale as a screening tool in clinical and community practice will assist in the targeted identification of women at higher risk for premature BF discontinuation. The generally low prevalence of breastfeeding among mothers in Cyprus and the absence of institutionalized breastfeeding community support programmes suggest the wider need for the design and evaluation of interventions beyond those focusing on the implementation of the Baby-friendly initiatives “10 steps” with a particular focus on theory-driven and researcher-informed community support interventions based on self-efficacy theory.

## Supplementary Information

**Additional file 1.** Questions about breastfeeding self-efficacy/confidence.

## Data Availability

The datasets generated or analyzed during the study are not publicly available because data analysis is still ongoing**.**

## Abbreviations

BF: Breastfeeding
BSES: Breastfeeding self-efficacy
BSES-SF: Breastfeeding self-efficacy short form
EBF: Exclusive breastfeeding
C/S: Cesarean Section
