# Supplementary material for: The association of breastfeeding self-efficacy with breastfeeding duration and exclusivity: longitudinal assessment of the predictive validity of the Greek version of the BSES-SF tool
Source: BMC Pregnancy Childbirth. 2021 Jun 9;21:421. doi: 10.1186/s12884-021-03878-3 (PMC8188677; doi:10.1186/s12884-021-03878-3)
Supplement: Supplementary file 1 — Additional file 1. Questions about breastfeeding self-efficacy/confidence. [file 12884_2021_3878_MOESM1_ESM.docx]

| **Ερωτήσεις σχετικά με την αυτοπεποίθηση για το θηλασμό** |
| --- |

Για κάθε μια από τις πιο κάτω προτάσεις, παρακαλώ να επιλέξετε την απάντηση που εκφράζει καλύτερα πόση σιγουριά - αυτοπεποίθηση νιώθετε όταν θηλάζετε το μωρό σας. *Είναι σημαντικό να προσπαθήσετε να απαντήσετε όλες τις ερωτήσεις σε αυτή την ενότητα, αφού με βάση τις απαντήσεις που θα δώσετε και στις 14 ερωτήσεις μπορεί να υπολογιστεί ένα συνολικό σκορ.*

Κυκλώστε τον αριθμό που εκφράζει περισσότερο το πώς αισθάνεστε, όπου…

καμιά σιγουριά

1= δεν αισθάνομαι καμιά σιγουριά

2= δεν αισθάνομαι και πολλή σιγουριά

μεγάλη σιγουριά

3= μερικές φορές αισθάνομαι σιγουριά

4= αισθάνομαι σιγουριά

5= αισθάνομαι μεγάλη σιγουριά

| 25. | Μπορώ πάντα να αξιολογώ ότι το μωρό μου παίρνει αρκετό γάλα | 1 | 2 | 3 | 4 | 5 |
| --- | --- | --- | --- | --- | --- | --- |
| 26. | Μπορώ πάντα να ανταπεξέρχομαι επιτυχημένα στο θηλασμό, όπως το έχω κάνει και με άλλες προκλήσεις | 1 | 2 | 3 | 4 | 5 |
| 27. | Μπορώ πάντα να θηλάζω το μωρό μου χωρίς να χρησιμοποιώ ξένο γάλα | 1 | 2 | 3 | 4 | 5 |
| 28. | Μπορώ πάντα να διασφαλίζω ότι το μωρό θηλάζει σωστά σε όλη την διάρκεια του θηλασμού | 1 | 2 | 3 | 4 | 5 |
| 29. | Μπορώ πάντα να τα καταφέρνω με την διαδικασία του θηλασμού ώστε να είμαι ικανοποιημένη | 1 | 2 | 3 | 4 | 5 |
| 30. | Μπορώ πάντα να καταφέρνω να θηλάζω ακόμη και όταν το μωρό μου κλαίει | 1 | 2 | 3 | 4 | 5 |
| 31. | Μπορώ πάντα να διατηρώ τη θέληση μου να θηλάζω | 1 | 2 | 3 | 4 | 5 |
| 32. | Μπορώ πάντα να θηλάζω άνετα όταν τα μέλη της οικογένειας μου είναι παρόντα | 1 | 2 | 3 | 4 | 5 |
| 33. | Μπορώ πάντα να είμαι ικανοποιημένη με την εμπειρία του θηλασμού | 1 | 2 | 3 | 4 | 5 |
| 34. | Μπορώ πάντα να διαχειρίζομαι το γεγονός ότι ο θηλασμός είναι χρονοβόρος | 1 | 2 | 3 | 4 | 5 |
| 35. | Μπορώ πάντα να ολοκληρώνω το θηλασμό με το ένα στήθος πριν αλλάξω στο άλλο | 1 | 2 | 3 | 4 | 5 |
| 36. | Μπορώ πάντα να συνεχίζω να θηλάζω σε κάθε γεύμα. | 1 | 2 | 3 | 4 | 5 |
| 37. | Μπορώ πάντα να ανταπεξέρχομαι στις ανάγκες του μωρού μου για θηλασμό | 1 | 2 | 3 | 4 | 5 |
| 38. | Μπορώ πάντα να καταλαβαίνω πότε το μωρό μου έχει σταματήσει να θηλάζει | 1 | 2 | 3 | 4 | 5 |
